# Retinoic Acid Restores Adult Hippocampal Neurogenesis and Reverses Spatial Memory Deficit in Vitamin A Deprived Rats

**DOI:** 10.1371/journal.pone.0003487

**Published:** 2008-10-22

**Authors:** Emilie Bonnet, Katia Touyarot, Serge Alfos, Véronique Pallet, Paul Higueret, Djoher Nora Abrous

**Affiliations:** 1 Nutrition & Neurosciences laboratory, University of Bordeaux 1, Talence, France; 2 University of Bordeaux 2, Bordeaux, France; 3 Neurogenesis & Pathophysiology laboratory, Bordeaux Neuroscience Research Center, INSERM 862, Bordeaux, France; Columbia University, United States of America

## Abstract

A dysfunction of retinoid hippocampal signaling pathway has been involved in the appearance of affective and cognitive disorders. However, the underlying neurobiological mechanisms remain unknown. Hippocampal granule neurons are generated throughout life and are involved in emotion and memory. Here, we investigated the effects of vitamin A deficiency (VAD) on neurogenesis and memory and the ability of retinoic acid (RA) treatment to prevent VAD-induced impairments. Adult retinoid-deficient rats were generated by a vitamin A-free diet from weaning in order to allow a normal development. The effects of VAD and/or RA administration were examined on hippocampal neurogenesis, retinoid target genes such as neurotrophin receptors and spatial reference memory measured in the water maze. Long-term VAD decreased neurogenesis and led to memory deficits. More importantly, these effects were reversed by 4 weeks of RA treatment. These beneficial effects may be in part related to an up-regulation of retinoid-mediated molecular events, such as the expression of the neurotrophin receptor TrkA. We have demonstrated for the first time that the effect of vitamin A deficient diet on the level of hippoccampal neurogenesis is reversible and that RA treatment is important for the maintenance of the hippocampal plasticity and function.

## Introduction

Vitamin A deficiency (VAD), leading to retinoic acid (RA) hyposignaling, represents a major public health problem and is estimated to affect 200 million children and adults in many countries [1], [2]. A disruption of retinoid signaling pathway has been involved in the pathophysiology of affective disorders, schizophrenia and late-onset Alzeimer's disease [2]–[8]. Animals' studies have shown that vitamin A and RA play a key role during brain development [9]–[12], and during adulthood, retinoids have been shown to modulate emotional and memory functions [2], [13].

The effects of retinoids on memory have been proposed to be mediated, at least in part, by an alteration of hippocampal plasticity. Indeed, retinoids are required for long term synaptic plasticity in the hippocampal formation (HF) [14], [15], a key structure in memory processing [16], [17]. Furthermore, vitamin A deficiency impairs spatial memory [18], [19]. In aged subjects, the naturally occurring hypoactivity of the retinoid signaling pathway also induces spatial memory and hippocampal long term potentiation deficits, which are alleviated by the normalization of brain retinoid signaling with RA treatment or nutritional vitamin A supplementation [20], [21]. Despite these striking relationships between retinoid signaling and memory, the mechanisms by which hippocampal retinoid hyposignaling influence learning abilities remain largely unknown.

The dentate gyrus (DG) of the HF is one of the areas where neurons are generated throughout the lifespan [22]–[24]. The newly born cells express neuronal markers, emit axons, receive synaptic inputs; in addition, their electrophysiological properties are very similar to those of mature dentate granule neurons. Neurogenesis has been hypothesized to play an important role in spatial memory [23], [25], [26]. Recently, its specific contribution to spatial memory evaluated in the water maze has been evidenced using genetic approaches [27], [28]. The ability of RA to promote *in vitro* neurogenesis [29]–[31] suggested that activation of retinoid signaling constitutes a therapeutic strategy to increase adult hippocampal neurogenesis and consequently hippocampal-dependent memory [3], [32]. However, contrasting results have been obtained *in vivo*. Long term exposure to RA decreases hippocampal neurogenesis [33], whereas maternal VAD disrupts irreversibly adult hippocampal neurogenesis in the adult offspring [34]. Consequently, the influence of retinoid signaling on this novel form of structural plasticity still remains controversial.

Here, we tested the hypothesis that retinoid hyposignaling decreases adult hippocampal neurogenesis and spatial memory. In order to address this issue, retinoid-deficient rats have been generated by a vitamin A-free diet from weaning. This nutritional approach enables normal embryogenesis and postnatal development, does not altered mother-infant interaction while permitting controlled vitamin A depletion in the adult rat. We further examined whether these effects could be reversed by RA treatment in adulthood. Finally, we investigated possible retinoid target genes as neurotrophin receptors that could be involved in these processes.We have demonstrated that both hippocampal neurogenesis and spatial memory can be rescued by RA treatment in vitamin A deficient rats.

## Materials and Methods

### Animals

Weaning male Wistar rats (3-week old) were purchased from Harlan (Gannat, France). They were housed two per cage in a room with a constant airflow system, controlled temperature (21–23°C), and a 12 h light/dark cycle. The rats were given *ad libitum* access to food and water and were randomly divided into two experimental groups. One group (n = 55) received a vitamin A-free diet (Laboratorio Piccionni, Italy), whereas the second group (n = 52) was fed with a control diet containing 5 IU retinol/g (INRA, Jouy en Josas). All animals were individually housed from one week prior to the beginning of RA treatment until sacrifice. All experiments were performed in accordance with the European Communities Council Directives (86/609/EEC) and the French national Committee (87/848) recommendations.

### Treatments

#### RA injections

Half the control and VAD rats were injected daily with RA (150 µg of *all-trans-*RA/kg, Sigma, France). RA was dissolved in a mixture (vehicle) containing polyethyleneglycol-NaCl-ethanol (70∶20∶10, by vol.). This dose of RA was shown to be effective in reversing age-related hypoexpression of brain signaling and its associated memory impairment [20]. The other half of the animals was treated daily with vehicle only.

#### 5-Bromo-2′-deoxyuridine (BrdU) injections

In order to label the newly born cells and examine hippocampal cell survival, BrdU, a thymidine analogue incorporated into genetic material during synthetic DNA phase of mitotic division, was used. Rats received a daily intraperitoneal injection of BrdU (50 mg/kg, Sigma, France), dissolved in phosphate buffer (0.1 M, pH 8.4), during the 4 consecutive days beginning 4 days after the first injection of RA.

### Immunohistochemistry

Rats were anesthetized with pentobarbital (100 µl per 100 g) and perfused transcardially with 200 ml of phosphate-buffered saline (PBS, pH 7.4) containing heparin, followed by 300 ml of 4% paraformaldehyde. After 1 week postfixation period in paraformaldehyde, 50 µm frontal sections were cut on a vibratome (Leica). Free-floating sections were processed with a standard immunohistochemical procedure [35]. A one–in-ten section was treated for KI-67 immunoreactivity using a mouse anti-KI-67 monoclonal antibody (1∶200, Novocastra, Newcastle, U.K.) or for double-cortin (DCX) immunoreactivity using a goat polyclonal antibody (1∶1000, Santa Cruz Biotechnology, Santa Cruz, California). Secondary antibodies were biotinylated horse anti-mouse and donkey anti-goat (1∶200, AbCys; 1∶200, Amersham). For BrdU labeling, adjacent sections were treated with 2N HCl to denature DNA (30 min at 37°C) and then washed in phosphate buffer. Sections were incubated with a mouse monoclonal anti-BrdU antibody (1∶200, Dako) followed by the biotinylated horse anti-mouse antibody (1∶200, AbCys). Sections were processed in parallel, and immunoreactivities were visualized by the biotin-streptavidin technique (ABC kit, Dako) by using 3,3′-diaminobenzidine as chromogen.

The number of immunoreactive (IR) cells in the left DG was estimated by using a modified version of the optical fractionator method with a systematic random sampling of every 10 sections along the rostrocaudal axis of the DG. On each section, IR cells in the granular and subgranular layers of the DG were counted with a 100× microscope objective [35]. All results are expressed as the total number of cells in the whole DG.

To analyze the phenotype of BrdU labeled cells, 8 rats per group were randomly selected. One in ten sections obtained from the second experiment was incubated with rat anti-BrdU monoclonal antibodies (1∶500, Servibio), which were revealed by using CY3-labeled anti-rat IgG antibodies (1∶1000, Interchim). Sections were then incubated with mouse monoclonal anti-NeuN antibodies (1∶1000, Euromedex), and bound anti-NeuN monoclonal antibodies were visualized with an Alexa 488 goat anti-rabbit IgG (1∶1000, Interchim). The percentage of BrdU-labeled cells that expressed NeuN was determined throughout the DG by using a confocal microscope with HeNe and Argon lasers (Nikon PCM 2000). All BrdU double labeled cells were examined, and sections were optically sliced in the Z plane by using a 1 µm interval. Cells were rotated in orthogonal planes to verify double labeling.

### Behavioral testing

Rats were tested in a Morris water maze (180 cm diameter, 60 cm high) filled with water (22°C) made opaque by addition of white paint. An escape platform was hidden 2 cm below the surface of the water in a fixed location in one of four quadrants halfway between the wall and the middle of the pool. Before the start of the training, animals were habituated to the pool without a platform for 1 min/day for 2 days. During training, animals were required to locate the submerged platform by using distal extramaze cues. They were tested for four trials per day (90 s with an intertrial interval of 60 s, beginning from three different start points randomized every day) for 7 consecutive days. The distance covered to find the platform and the time to reach the platform were measured with a computerized tracking system (Videotrack, Viewpoint, Lyon, France). After the last training day, on day 8, animals were placed for 60 s in the pool without the platform (probe test). Performance was evaluated by the percentage of time spent in the quadrant where platform was located during training (target quadrant). Finally, in order to control for visual acuity deficits, the hidden platform was replaced by a visible platform located in the opposite quadrant, and animals were tested for four trials (90 s) over one day. One control rat treated with vehicle was excluded from the experiment due to failure to search for the platform during the acquisition phase (tigmotaxis).

### Real-Time PCR analysis of neurotrophin receptor expression

Rats were sacrificed by decapitation, and each hippocampus was rapidly removed and stored at −80°C in order to measure neurotrophin receptor expression. Extraction of RNA was conducted using an extraction kit (TRIzol reagent, Invitrogen, France) according to the manufacturer's instructions. The quality and the concentration of RNA were determined by spectrophotometry. Then, the integrity of the purified RNA was verified using the RNA 6000 Pico LabChip kit in combination with the 2100 bioanalyser (Agilent Technologies). Using OligodT and random primers (Promega, France), cDNA was synthesized with ImPromII reverse transcriptase (Promega, France). Briefly, 1 µg of total RNA mixed with RNasin (Promega, France) and DNase (Roche, France) was incubated at 37°C. Then, OligodT plus random primers were added for incubation at 70°C. The reverse transcriptase reaction was performed at 42°C for 60 min in a final volume of 20 µl. The polymerase chain reaction (PCR) was performed in a LightCycler system (Roche Diagnostics, Germany). The forward and reverse primer sequences for each gene are in Table 1. To detect target genes amplification products, a LightCycler DNA Master SYBR Green I kit was used according to the manufacturer's instructions. PCR was performed in micro-capillary tubes in a final volume of 20 µl, containing 1× LC-DNA Master Green I mix, 4 mM MgCl_2_, 0.5 µM of each primer and 4 µl cDNA. The specificity and the identity of the amplified products was verified as follows: (1) melting curve analysis showed a single melting peak after amplification, and (2) amplified products for each gene were verified by sequencing with the Big Dye Terminator v1.1. (Applied Biosystems) and analyzed on a ABI 3130 sequencer (Applied Biosystems).

**Table 1:** Primers used for Light Cycler RT-PCR.

Gene name	Nucleotide sequence	Product lenght (bp)
**PPIB**	**F: 5′-GTTCTGGAAGGCATGGATGT-3′** **R: 5′-TCCCCGAGGCTCTCTCTACT-3′**	**153**
**BMG**	**F: 5′-GCCCAACTTCCTCAACTGCTACG-3′** **R: 5′-GCATATACATCGGTCTCGGTGGG-3′**	**180**
**TrkA**	**F: 5′-ACTGGGTGGCAGTTCTCTTTCC-3′** **R: 5′-TCCTGGCGCTTGATATGGTG-3′**	**117**
**TrkB**	**F: 5′-TTCCGGTGGTTTTAGCCTGTG-3′** **R: 5′-TCACTCCTGCTGTGCTTTATGG-3′**	**122**

Sequences are shown for foward (F) and reverse (R) primers. PPIB: peptidylprolyl isomerase B (cyclophilin B); BMG: β2-microglobulin, TrkA: tropomyosin-related kinase A; TrkB: tropomyosin-related kinase B.

Quantification data were analyzed using the LightCycler Relative Quantification Software (Roche, Germany). Due to the fact that target and reference genes have different sequences and amplicon lengths, different PCR efficiencies could be found. For this reason, the software provides a calibrator-normalized relative quantification including a PCR efficiency correction [36]. In our case, the calibrator was chosen among the control rats. Results are expressed as the target/reference ratio divided by the target/reference ratio of the calibrator. Two housekeeping genes, PPIB and BMG, were used to quantify the expression of each gene (i.e TrkA, TrkB) in order to avoid possible errors related to our practice of using only one reference gene for normalization [37]. Thus, the expression of these housekeeping genes, which is the same in all groups of animals, has been shown to be unaffected by our experimental conditions. The results presented are normalized in comparison to PPIB.

### Measurement of serum retinol concentration

Blood was collected and spun at 3000 rpm for 15 minutes. The supernatant was removed and snap frozen on dry ice. Serum retinol was assayed by HPLC according to a previously described method [38].

### Experimental design

#### First experiment: effects of 11 weeks of VAD and one week RA treatment (between the 10^th^ and 11^th^ week of VAD) on neurogenesis

**Figure 1:**
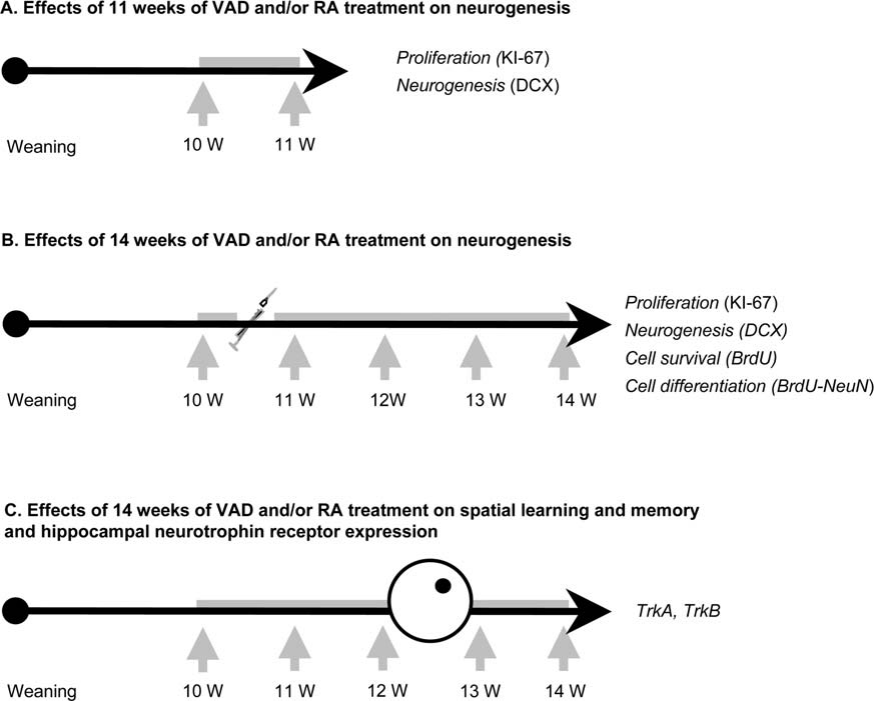
Experimental protocols. Weaning rats (3 weeks old) were submitted to 11 weeks or 14 weeks of vitamin A deficiency (VAD). The first two experiments were intended to study the effects of VAD and/or RA administration on hippocampal neurogenesis. The third experiment was designed to study the effects of VAD and/or RA administration on spatial memory and hippocampal neurotrophic receptor expression. The arrows and the grey bars indicate VAD and RA treatment, respectively.

We examined the effects of 11 weeks of a vitamin A-free diet on cell proliferation and neurogenesis in the DG. In order to study the role of RA, control and VAD rats were injected with RA or vehicle daily for one week during the 10^th^ week of VAD. All groups (Control+vehicle, n = 8; Control+RA, n = 8; VAD+vehicle, n = 8; VAD+RA n = 9) were sacrificed at the 11^th^ week of VAD (Fig. 1A). Cell proliferation was examined using an endogenous marker of the cell cycle, KI-67. DCX was used as a surrogate of neurogenesis.

#### Second experiment: effects of 14 weeks of VAD and four weeks RA treatment (between the 10^th^ and 14^th^ week of VAD) on cell survival and differentiation

In a subsequent experiment, we examined the effects of VAD and RA treatment on cell survival and differentiation. From the 10^th^ week of VAD, animals were injected with RA or vehicle daily for four weeks (Control+vehicle, n = 9; Control+RA, n = 8; VAD+vehicle, n = 9; VAD+RA n = 9). Four days after the beginning of RA treatment, all groups were injected with BrdU for 4 days. Rats were allowed to survive for another three weeks after the last injection of BrdU and continued treatment in their respective experimental conditions (Fig. 1B). In order to obtain more information about adult neurogenesis independent of BrdU, we studied the expression of DCX. Cell proliferation was also studied using the endogenous marker, KI-67.

#### Third experiment: effects of 14 weeks of VAD and four weeks RA treatment (between the 10^th^ and 14^th^ week of VAD) on spatial learning and memory and hippocampal neurotrophin receptor expression

We then examined the influence of VAD and RA treatment on spatial memory. From the 10^th^ week of VAD, animals were injected with RA or vehicle (Control+vehicle, n = 9; Control+RA, n = 10; VAD+vehicle, n = 10; VAD+RA, n = 10). Two weeks later, animals were tested in a watermaze task. All groups were sacrificed one week after the completion of behavioral testing to analyze neurotrophin receptor expression (Fig. 1C).

### Statistical analysis

All results were expressed as mean±SEM. Data were submitted to analyses of variance. When appropriate, post-hoc comparisons were performed using the Fisher PLSD test. Whenever two groups were compared, an unpaired t-test was used.

## Results

### Status of vitamin A deficiency

Analysis of serum retinol levels was performed after 11 or 14 weeks of VAD in order to confirm the status of VAD rats. Serum retinol concentration was significantly diminished by 11 weeks of VAD [Control:1.08±0.07 µmol/l; VAD:0.07±0.006 µmol/l, t_(14)_ = −10.45, p<0.0001]. However, a whole vitamin A depletion was produced by 14 weeks of VAD, retinol being undetectable in the VAD serum at that time [Control:1.46±0.08 µmol/l; VAD:<0.01 µmol/l].

### Effects of vitamin A deficiency and RA treatment on hippocampal neurogenesis

**Figure 2:**
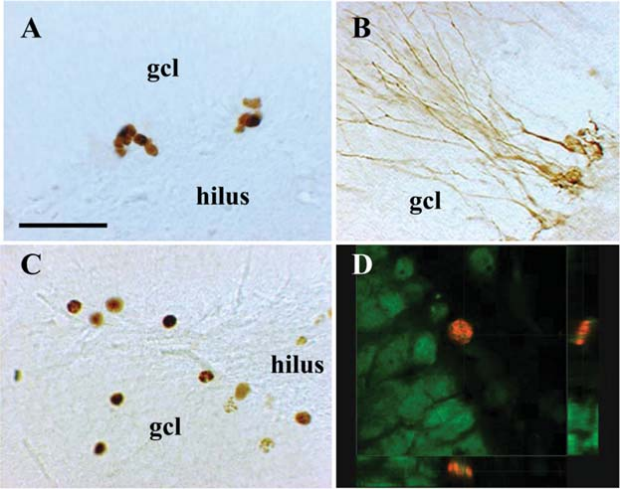
Illustration of neurogenesis in the dentate gyrus. Examples of : (A) Ki67-IR cells (B) DCX-IR cells and (C) 3-week-old BrdU-IR cells. (D) Three dimensional reconstruction of a z series along the y-z axis (narrow right panel) and x-z axis (narrow bottom panel) showing that a 3-week-old newly born cell (red) is double stained with the neuronal marker NeuN (green). Scale bar : A–C, 50 µm. gcl = granule cell layer.

**Figure 3:**
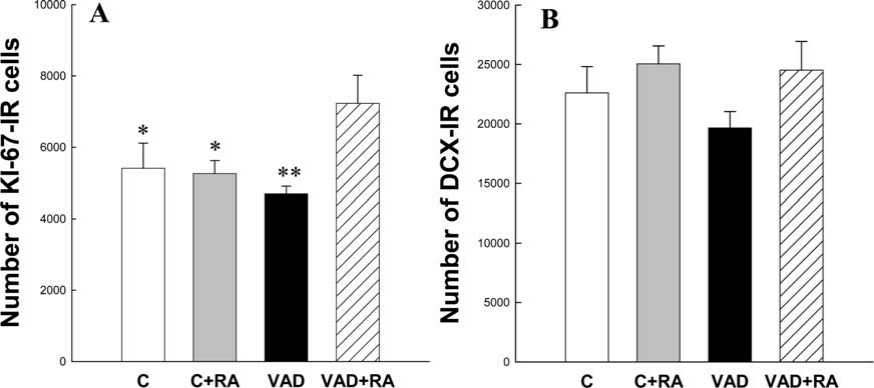
Effects of 11 weeks of vitamin A deficiency and RA treatment on hippocampal neurogenesis. Total number of: (A) KI-67-IR cells and (B) DCX-IR cells in the DG after 11 weeks of VAD. VAD for 11 weeks does not affect cell proliferation or the number of immature DCX neurons.*p<0.05, **p<0.01 when compared to VAD+RA.

The influence of VAD and RA treatment were examined on hippocampal cell proliferation and neurogenesis (experiment 1). Cell proliferation was measured following 11 weeks of VAD, using an endogenous marker of cell cycle, KI-67 [39]. KI-67-labeled cells were located within the subgranular zone and were isolated or grouped in clusters (Fig. 2A). Quantitative analysis revealed that neither a control diet with or without RA injections, nor a VAD diet alone had an effect on cell proliferation (Fig. 3A). In contrast, the number of KI-67 expressing cells was increased by ∼35% in VAD rats receiving RA injections for one week [F_(3,29)_ = 3.66,p<0.05;C = C+RA = VAD<VAD+RA at least p<0.05]. We also determined whether 11 weeks of VAD and 1 week of RA treatment influence neurogenesis by using double-cortin (DCX), a microtubule-associated phosphoprotein, as a surrogate [40]. DCX-IR cells were located in the deepest region of granule cell layer (gcl) at the interface of the hilus. Their dendrites radiated into the molecular layer (Fig. 2B). A quantitative analysis revealed that 11 weeks of VAD or RA treatment has no effect on the number of newly generated neurons [Fig. 3B, F_(3,29)_ = 1.51,p = 0.23].

The aim of the second study was twofold: (1) to determine whether cell proliferation and neurogenesis were influenced by a longer vitamin A deficient diet and RA treatment, and (2) to determine whether the survival and differentiation of cells born during the 10^th^ week of VAD were influenced by subsequent VAD and/or RA treatment. To address this issue, animals were injected with BrdU 10 weeks after the beginning of VAD and allowed to survive for 3 additional weeks.

**Figure 4:**
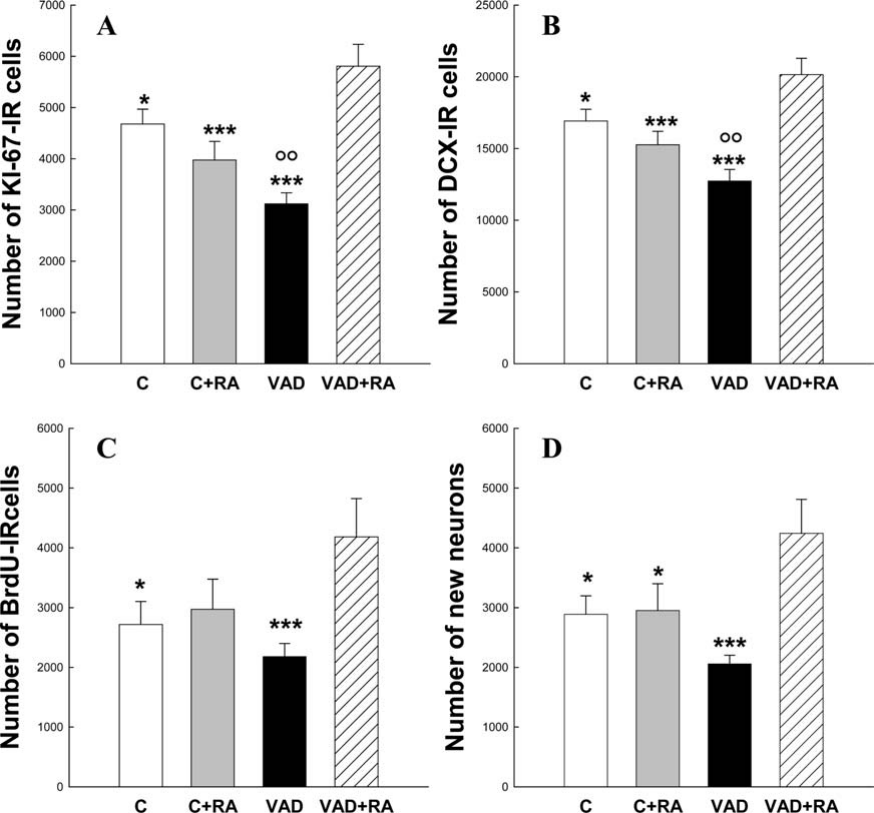
Effects of 14 weeks of vitamin A deficiency and RA treatment on hippocampal neurogenesis. Total number of : (A) KI-67-IR cells, (B) DCX-IR cells, (C) 3 weeks old BrdU-IR cells and D) the extrapolated number of newly born neurons after 14 weeks of VAD. VAD for 14 weeks decreases cell proliferation and neurogenesis, an effect reversed by 4 weeks RA treatment.*p<0.05; ***p<0.001 when compared to VAD+RA, °°p<0.01 when compared to C.

As expected, after 14 weeks, VAD decreased cell proliferation by ∼32%. This effect was completely overcompensated by RA treatment, which by itself did not have any effect in control animals [Fig. 4A, F_(3,31)_ = 11.95,p<0.0001 with VAD<C = C+RA<VAD+RA at least p<0.05]. We also found that the number of DCX expressing cells was decreased in VAD rats by ∼25%, and this effect was overcompensated by RA administration [Fig. 4B; F_(3,31)_ = 11.40,p<0.0001 with VAD<C = C+RA<VAD+RA at least p<0.05].

When examining the effect of VAD and/or RA on cell survival, we found that most of the 3-week-old surviving BrdU-IR cells were isolated, round, large and located within the gcl (Fig. 2C). As shown in Fig. 4C, the number of BrdU-IR cells was not affected by a control diet with or without RA injections nor a VAD diet alone. In contrast, the number of BrdU labeled cells in VAD rats injected with RA was greater than that measured in the other groups [F_(3,31)_ = 3.46,p<0.05 with C = VAD = C+RA<VAD+RA at least p<0.05].

The phenotype of newly born cells labeled with BrdU was determined using NeuN, a neuronal marker. The percentage of BrdU/NeuN double stained cells located in the gcl (Fig. 2D) did not differ between the four experimental groups [C:92.9±1.4, C+RA:91.4±1.4, VAD:89.8±2.3, VAD+RA:94.5±1.8; F_(3,28)_ = 1.23,p = 0.31]. The ratio of BrdU-IR cells colabeled with NeuN was multiplied by the total number of BrdU-labeled cells to give an estimate of the total number of BrdU-labeled neurons. The extrapolated total number of 3-week-old, BrdU-labeled neurons in VAD rats receiving RA injections was higher than that of the other groups [Fig. 4D, F_(3,28)_ = 5.162,p<0.01; with C = VAD = C+RA<VAD+RA at least p<0.05]. We then calculated the rate of cell survival by comparing, within each animal, the number of 3-week-old BrdU-IR cells to the number of proliferation KI67 cells. We found that this ratio was similar among the different groups [C:0.57±0.07, C+RA:0.75±0.10, VAD:0.72±0.10, VAD+RA:0.73±0.11, F_(3,31)_ = 0.64, p = 0.59].

Thus, altogether these results showed that VAD decreases cell proliferation and neurogenesis; these effects are reversed by 4 weeks RA treatment. In contrast, cell survival and cell differentiation are not influenced by VAD and/or RA treatment.

### Effects of vitamin A deficiency and RA treatment on spatial learning and memory

**Figure 5:**
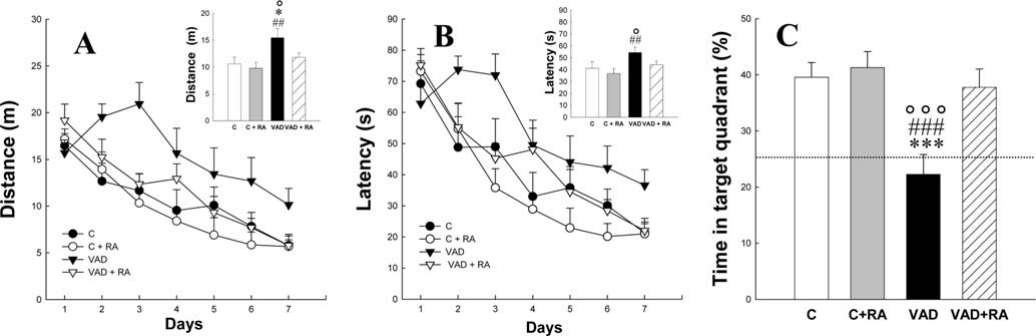
Effects of 14 weeks of vitamin A deficiency and RA treatment on spatial memory in the water maze. Spatial learning as shown by the evolution of the mean distance (A) covered by rats or the latency (B) to find the hidden platform. In the insert are shown the mean distance or the mean latency over the seven days of training. (C) Percentage of time spent by rats in the target quadrant; the dotted line corresponds to chance level. VAD-induced spatial memory deficits are rescued by RA treatment. ##p<0.01, ###p<0.001 when compared to C+RA, °p<0.05, °°°p<0.001 when compared to controls, *p<0.05, ***p<0.001 when compared to VAD+RA.

The previous experiments suggested that neurogenesis, as evaluated with DCX, was impaired after 11 weeks of VAD. Indeed, immature neurons expressed DCX until they are 2–3 weeks old [40], [41]. The 3 weeks delay necessary to observe a decrease in DCX expression indicates that reduction in cell proliferation occurred between the 11^th^ and the 12^th^ week of the VAD. For this reason, animals were trained in the water maze between the 12^th^ and 13^th^ week (Fig. 1C). In the water maze, animals are required to locate a hidden platform using the spatial cues available in the testing room. Control animals and animals treated with RA learned this task as shown by the progressive decrease in the distance covered to reach the hidden platform over the seven days of training (Fig. 5A). Memory impairment was observed in VAD rats that traveled a higher distance to find the platform. This deficit was reversed in VAD rats receiving RA treatment, with performance being similar to that observed in control rats [F_(3,35)_ = 4.075,p<0.05 with VAD>C = C+RA = VAD+RA at least p<0.05]. Similar results were obtained for the latency to find the hidden platform [Fig. 5B, F_(3,35)_ = 3.12,p<0.05 with VAD>C = C+RA = VAD+RA at least p<0.05]. VAD rats exhibited normal motor functioning, as evidenced by the lack of a significant difference in swimming speed [data not shown, F_(3,35)_ = 1.32,p = 0.28].

On day 8, memory for the platform location was tested using a probe test. The time spent in the quadrant previously containing the platform was measured. VAD rats failed to display a memory for the platform location, as indicated by a percent time swimming in the target quadrant around the chance level (25%, Fig. 5C). This deficit was reversed by RA administration [F_(3,35)_ = 8.076,p<0.001; with VAD<C = C+RA = VAD+RA at least p<0.01]. After the probe trial on day 9, animals were trained to find a visible platform. The distance traveled [F_(3,35)_ = 3.35,p = 0.093] and the latency [F_(3,35)_ = 1.85,p = 0.15] to find a visible platform were identical for the different groups. These results indicate that learning differences were not due to differences in motor or visual capabilities, thigmotaxic behavior, or more generally to differences in health status.

Taken together, these results showed that VAD induced spatial memory deficits in the water maze that could be reversed by RA administration.

### Effects of vitamin A deficiency and RA treatment on hippocampal neurotrophin receptor expression

**Figure 6:**
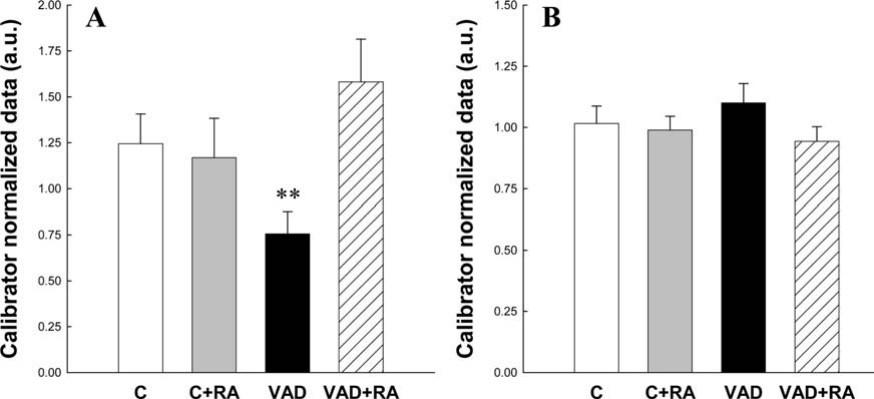
Effects of 14 weeks of vitamin A deficiency and RA treatment on mRNA expression of neurotrophic receptors in the hippocampus. (A) TrkA and (B) TrkB mRNA expression as quantified by Real Time-PCR. RA treatment compensated VAD-induced reduction in hippocampal TrkA mRNA.**p<0.01 when compared to VAD+RA.

The ability of RA to promote neurogenesis and improve memory abilities in VAD rats could be in part mediated by activation of neurogenesis-related gene expression via neurotrophin receptors, which are known to be regulated by RA *in vitro* [30], [42]–[46]. To uncover the possible mechanisms involved in the effect of VAD and RA treatment on neurogenesis, animals were sacrificed one week after the behavioral experiment (Fig. 1c). As seen in Fig. 6A, quantitative analysis of hippocampal TrkA mRNA expression indicated differences between groups [F_(3,34)_ = 3.07,p<0.05]. Indeed, we observed that VAD tended to reduce hippocampal TrkA mRNA expression compared to control rats (36%, p = 0.09), which is fully upregulated by RA treatment (VAD<VAD+RA, p = 0.01). In contrast, RA administration in control rats had no effect on TrkA mRNA expression. When considering hippocampal levels of TrkB mRNAs, no significant variation was observed between groups [Fig. 6B, F_(3,34)_ = 0.98, p = 0.41].

These data suggested that RA signaling may regulate TrkA transcription in the hippocampus that could be an important regulatory mechanism involved in the restoration of adult neurogenesis and spatial memory in VAD rats.

## Discussion

The results of the present experiments show that 14 weeks of VAD decreases hippocampal neurogenesis, based on the numbers of doublecortin-IR cells, and impairs spatial memory. These effects are reversed by 4 weeks RA treatment. Furthermore, the restoration of neurogenesis in VAD rats receiving RA treatment may in part be related to up-regulation of retinoid-mediated molecular events, such as the expression of the neurotrophin receptor TrkA.

We have shown that a VAD starting at weaning for 11 weeks did not affect cell proliferation or the number of immature DCX neurons when compared to control animals fed with a control diet containing 5 IU retinol/g. Although serum retinol concentration, a good indicator of vitamin A depletion, was significantly reduced, 11 weeks of diet was not sufficient to entirely deplete vitamin A reservoir. This may explain the lack of effects on neurogenesis. This result is in line with a previous study failing to observe a down-regulation of RA-regulated genes within the hippocampus after a short-term VAD (10 weeks). That treatment, however, was sufficient to decrease target gene expression in the striatum [47]. In contrast, a total depletion in serum retinol levels was observed after 14 weeks of VAD (undetectable levels). In this condition, we observed a decrease in cell proliferation and neurogenesis, as indicated by changes in KI-67 and DCX. This cytoplasmic protein is expressed by immature neurons until they are 2–3 weeks old [40], [41]. This developmental time course, together with the 3 weeks delay necessary to observe a decrease in DCX expression, suggests that the loss in immature neurons results from an initial reduction in cell proliferation occurring after the 11^th^ week of the VAD. Furthermore, VAD did not seem to influence cell survival and differentiation. Indeed, the survival of the cells born during the 10^th^ week of the VAD was not impaired by additional 4 weeks of VAD, and the rate of survival calculated in the same animals was not influenced by VAD. These results contrast with those obtained recently, which show that VAD administration from birth to 18 weeks of age failed to influence cell proliferation while decreasing the survival and neuronal differentiation of 3-week-old newly born cells [34]. The discrepancy between the two studies may be related to differences in the animal models. Indeed, in our study VAD was begun at weaning sparing the early postnatal period whereas in the other study VAD was began from birth. Differences in the duration of the vitamin A deficient diet, and/or the time and method of RA supplementation could also be involved.

Administration of RA (*all-trans*) to control rats for one or four weeks did not modify neurogenesis. This contrasts with a previous study showing that *13–cis-*RA (anti-acne drug accutane) decreases hippocampal neurogenesis in mice after 3 weeks of treatment [33]. Species differences in RA sensitivity and/or differences in the dose of RA (150 µg vs 1 mg/kg/day) may explain the discrepancy between these studies. Furthermore, because *13-cis* RA has a low affinity for RA receptors [48], the biological effects of these two retinoic acid isomers may also differ.

More importantly, we found that RA was very potent in animals with a RA hypo-signaling. First, it increased cell proliferation in rats submitted to 11 weeks of VAD. Second, the supernumerary cells generated in animals submitted to 11 weeks of VAD survived and differentiated into neurons. Third, 4 weeks RA treatment to 14-week-old VAD rats increased cell proliferation and neurogenesis (i.e. number of DCX neurons and number of BrdU-NeuN co-labeled neurons) above the control values. This overcompensation might be related to a hypersensitivity of the molecular cascade downstream the RA receptors (see below). In line with these results, neonatal administration of an inhibitor of RA synthesis (disulfiram) decreased cell proliferation in the subventricular zone (SVZ), another neurogenic zone [31].

RA may regulate neurogenesis via several mechanisms. RA might directly regulate neurogenesis by acting through its specific nuclear receptors, the nuclear retinoic acid receptors (RAR_α,β,γ_) and the retinoid X receptors (RXR_α,β,γ_) [49]–[51], which are expressed by immature dividing cells. In the adult SVZ, a population of slowly dividing cells, the stem cells, has been shown to be activated by RA [52]. Consistent with that finding, SVZ–derived neurospheres expressing RAR_α,β,γ_ receptors also depend on RA signaling [52]. Thus, RA might increase hippocampal neurogenesis by activating the proliferation of stem cells present in this area. Moreover, RA has been shown to regulate neurogenesis *in vitro* by activating neurogenesis-related gene expression, including neurotrophin receptors [30]. Thus, the effects of VAD and RA were examined on TrkA, a receptor for Nerve Growth Factor (NGF) [42], [45], [46], and TrkB, a receptor for Brain Derived Neurotrophic Factor [43], [44] known to be expressed in the HF [53], [54]. Our results showed that VAD tended to reduce hippocampal TrkA expression (difference that was statistically significant when comparing TrkA expression between control and VAD groups using a t Test, p = 0.028). This effect was reversed by RA administration. In contrast, TrkB was not modified by VAD or RA. This finding suggested that RA, acting by increasing hippocampal expression of TrkA receptors, can potentiate NGF/TrkA signaling. This signaling may sustain the RA-induced increase in neurogenesis in VAD rats. Another non-exclusive possibility involves an indirect action *via* the septo-hippocampal cholinergic pathway. VAD reduces the activity of this pathway [18], [55], known to be under the control of NGF [56], [57]. Furthermore, immunolesion of this pathway decreases hippocampal neurogenesis [58], [59], whereas chronic treatment with NGF increases hippocampal neurogenesis [60]. Thus, it is possible that VAD impairs neurogenesis via a downregulation of the septo-hippocampal cholinergic pathway and that RA restores neurogenesis via an increased activity of these neurons.

Our results also demonstrated that VAD induced deficits in spatial memory in the water maze. Memory deficits evidenced in VAD rats did not result from visual alterations or motor impairments, known to appear following long-term diet [61]–[63] as they were able to find a visible platform. Furthermore, VAD rats were able to swim at similar speeds as control rats. The decline in spatial memory in VAD rats was fully restored by the RA administration, suggesting that activation of retinoid signaling through RA nuclear receptors is sufficient to alleviate the symptoms. Previous studies have shown that VAD [18], [19] or age-related brain retinoid hyposignaling [20] impairs spatial memory. More controversial are the effects of RA that can alleviate in some cases [18], [20] but not in all [19] retinoid hyposignaling-induced memory deficits. Furthermore, chronic 13*-cis* RA treatment of rats given a normal diet has been shown to either induce spatial memory deficits [33] or to produce no effect [64]. This discrepancy may be related to differences in subject age, species, and treatment used. In one study, spatial learning was impaired following a chronic RA treatment [33], a deficit probably due to the non-physiological dose of RA used (1 mg/kg).

Altogether, the present results suggest that spatial memory deficits observed after a hypoactivity of retinoid signaling could be in part related to an alteration of hippocampal neurogenesis. This contention is supported by the fact that RA treatment in VAD rats restores both hippocampal neurogenesis and hippocampal-dependent memory. Our VAD rats were profoundly impaired in the acquisition of spatial memory and exhibited the same learning curve as transgenic mice with ablation of adult-born hippocampal neurons [65]. However, future studies are needed to confirm a causal relationship between VAD-induced changes in neurogenesis and spatial memory. Our results also show that RA regulates neurogenesis and memory function by activating the transcription of TrkA receptors. However, we cannot exclude the possibility that a change in retinoid signaling influences neurogenesis and memory through a modification of synaptic plasticity. Indeed, VAD results in a reversible loss of hippocampal CA1 long term potentiation (LTP) and long term depression (LTD) [15]. Furthermore, age-related hypoactivity of retinoid signaling pathway impairs CA1 LTP, an effect abrogated by the normalization of retinoid signaling [20]. Thus, VAD-induced changes in synaptic plasticity within the DG could alter neurogenesis and spatial memory. This hypothesis is supported by the observation that hippocampal neurogenesis is increased by LTP [66]. However, controversial results have been obtained on the link between neurogenesis and LTP [67], [68] indicating that we cannot exclude that RA signaling affects hippocampal functions and neurogenesis through other mechanisms. Interestingly, memory dysfunction in aged rats, associated with hippocampal retinoid hyposignaling, is alleviated by RA-induced normalization of this retinoid signaling pathway [20]. Given that memory abilities have been related to hippocampal neurogenesis in aged rats [69], [70], this raises the issue as to whether RA-induced improvement in memory function in aged subjects depend upon an enhancement of neurogenesis.

Taken together, these data highlight the role of RA signaling in hippocampal plasticity and function. This is the first study showing that RA treatment, can counteract the effects of vitamin A deficiency on adult hippocampal neurogenesis disruption, one of the plasticity mechanisms involved in hippocampal-dependent spatial memory. Given the likely effects of RA treatment on hippocampus plasticity and function, a number of important future approaches arise from these results. In particular, the involvement of retinoids as a valuable strategy for the treatment of hippocampal-dependent disorders by promoting hippocampal plasticity and neurogenesis should be investigated.
